# Narrative and Bodily Identity in Eating Disorders: Toward an Integrated Theoretical-Clinical Approach

**DOI:** 10.3389/fpsyg.2021.785004

**Published:** 2021-12-15

**Authors:** Rosa Antonella Pellegrini, Sarah Finzi, Fabio Veglia, Giulia Di Fini

**Affiliations:** ^1^Centro Clinico Crocetta, Scuola di Specializzazione in Psicoterapia Cognitiva, Turin, Italy; ^2^Department of Psychology, University of Turin, Turin, Italy

**Keywords:** eating disorders, attachment, identity, somatic memory, narrative memory

## Abstract

Eating disorders (EDs) can be viewed as “embodied acts” that help to cope with internal and external demands that are perceived as overwhelming. The maintenance of EDs affects the entire identity of the person; the lack of a defined; or valid sense of self is expressed in terms of both physical body and personal identity. According to attachment theory, primary relationships characterized by insecurity, traumatic experiences, poor mirroring, and emotional attunement lead to the development of dysfunctional regulatory strategies. Although the literature shows an association between attachment style or states of mind, trauma, behavioral strategies, and various EDs, the debate is still ongoing and the results are still conflicting. Therefore, we believe it is important to examine and treat EDs by understanding which narrative trajectory intercepts distress in relation to narrative and embodied self-concept. Drawing on clinical observation and a narrative review of the literature, we focus on the construction and organization of bodily and narrative identity. Because bodily representations are the primary tools for generating meaning, organizing experience, and shaping social identity from the earliest stages of life, we focus on the role that bodily interactions and sensorimotor and proprioceptive patterns have played in the development of EDs. We consider the role that lack of attunement, insecure attachment, and relational trauma play in mentalizing, affecting self-representation and emotion regulation strategies. The paper also considers a semantic mode of trauma in EDs that involves a top-down pathway through beliefs and narratives about oneself based on lack of amiability, on devaluation, and on humiliation memories. Finally, we would like to highlight the proposal of an integrated model with multiple access model to psychotherapy that takes into account the complexity of ED patients in whom aspects related to dysregulation, body image disintegration, and post-traumatic symptoms are associated with a suffering sense of self and a retraumatizing narrative.

## Introduction

Anorexia nervosa (AN), bulimia nervosa (BN), binge eating disorder (BED), and other eating disorders (EDs) are characterized by poor awareness and emotional regulation in the form of “embodied acts” used to cope with internal and external demands that are perceived as overwhelming and stressful (Cook-Cottone, 2006; Verschueren et al., 2021). In some cases, self-assessment is dependent on body shape and weight, leading to persistent dissatisfaction and concerns about one’s worth and body image. The comparison between one’s own body and the bodies of others, and the obsessive search for imperfections that need to be corrected in order to achieve unrealistic standards, increases the discrepancy between the real body and the ideal body in a vicious cycle, thus increasing dissatisfaction. In other patients with ED, over-control of the body is functional for coping with anxiety or post-traumatic symptoms, independent of problems related to bodily dissatisfaction.

The maintenance of EDs involves the entire identity of the person; the lack of a definite; or valid sense of self is expressed in terms of both physical body and personal identity. In fact, many unhealthy eating behaviors are functional to maintaining the sense of self and a coherence of the patient’s belief system, to feeling special or to perceiving control and protection. Instead of evaluating themselves and building their own identity on the basis of different domains of life, people evaluate themselves mainly on the basis of the control they are able to exercise over their body and nutrition.

The individual builds his or her own bodily self during the pre-linguistic stage, in early interactions with the caregiver, and in the progressive differentiation between self and other through the encoding of sensory stimuli. Given the close relationship between somatosensory processing and the formation of the self-concept demonstrated in studies of embodied cognition (Meteyard et al., 2012) and in recent neuroimaging studies (Ronga et al., 2021), the purpose of this article is to examine the relationship between somatic and narrative memory from the perspective of identity construction early in life. Since bodily representations are the main tools for the generation of meaning, the organization of experiences, and the shaping of social identity, the first part of the article discusses the connection between somatic memory, narrative memory, identity construction, and eating disorders. In particular, we investigate the role of the first bodily interactions and the formation of sensorimotor and proprioceptive patterns in the development of EDs. The acquisition of language and of a more articulated ability to represent reality allow the individual to develop a narrative self that, as in individuals with an ED, often expresses the sufferance on some critical themes, such as a sense of low personal value and a lack of amiability (Veglia and Di Fini, 2017), accompanied by the feeling of never being adequate in relationships (and up to chronic shame). Thus, it is important to also consider the narrative function of the body and the relationship to one’s body boundaries when examining EDs.

Given the role of early attachment relationships in the development of EDs, and based on an ongoing debate about the prevalence of attachment styles and states in individuals with ED, the second part of the article focuses on the dynamics communicated through the body and/or the eating behaviors in ED. The reflection we describe draws its main ideas from clinical observations, clinical practice, and the study of the theoretical and empirical literature. Specifically, we propose a possible functional model that considers the impact of early relational trauma on hetero-regulation and self-regulation skills and strategies, changing strategies for maintaining attachment relationships, and the parallel unfolding of EDs, even as they transition from one set of symptoms to another over time. In line with many studies (Palmisano et al., 2016; Pignatelli et al., 2017; Monteleone et al., 2020) the focus is also on the possible links between adverse childhood experiences and the development of EDs. Indeed, according to attachment theory (Bowlby, 1973), primary relationships characterized by insecurity, poor mirroring, and poor emotional attunement (Tronick, 1989) favor the development of dysfunctional regulatory strategies, which in turn may be a risk factor for ED development.

The literature has highlighted the high incidence of negative and traumatic experiences in the histories of people with EDs (Tasca et al., 2013) and reported a prevalence of emotional neglect (Pignatelli et al., 2017) that does not allow for optimal development of the ability to read and differentiate one’s emotional states or integrate them with physical states. Thus, they remain separate and no longer represent a source of self-awareness. Given the role of early relational trauma as a vulnerability factor in the development of ED, as well as the fundamental importance of semantic self-representation in the maintenance of these disorders, the third section of the article proposes a typology of traumatization that incorporates a top-down approach involving the role of semantic and narrative memory in the formation of negative self-representations and beliefs. This part then moves the reflection on the level of the main intrapersonal dynamics that are communicated through the ED.

In terms of clinical implications, the fourth part of the article aims to highlight the proposal of an integrated model with multiple approaches to psychotherapy (Veglia et al., 2019) that takes into account the complexity of the patient with ED, his particular way of attributing meaning and his preferred ways (procedural, verbal-declarative, or affective-relational) of processing the experiences.

## Connection Between Somatic Memory, Narrative Memory, Identity Construction, and Eating Disorders

The socio-emotional ontogenesis of the child develops in close relation to the maturation of its sensory systems. In particular, the first year of life is studded with somatosensory experiences that play an essential role in emotional and social development (Wright, 1991). Sensory channels, such as the visual pathway, function as an interpersonal communication channel that allows for the transmission of reciprocal influences (Schore and Schore, 2008). The interaction between the child and his caregiver develops as a kind of speechless dialog (Spitz, 1958), characterized by mutual affective mirroring modulated by a continuous synchronization of affects in which the two members of the dyad engage each other through the coordination of affective responses.

It has been observed that the child looks away after a visual interaction characterized by a high affective level. This indicates the need to regulate the potentially disorganizing effect of an overly intense emotion, such as a look of joy or anger (Fogel, 1982). If the caregiver is well adjusted, he or she can understand what is happening and allows the child the necessary space to self-regulate by withdrawing and waiting for the signals that indicate the child’s readiness for new involvement. Mirroring is characterized by interactions with continuous regulatory mechanisms (autonomous and relational); thus, emotional regulation develops within this synchronized mode of interaction (Schore and Schore, 2008).

The moments of mirroring trigger changes in the mental states of both the mother and the infant and, in the case of attunement, allow for the re-establishment of a mutually regulating activation system (Beebe and Lachmann, 1988). Therefore, attachment relationships characterized by intrusiveness or lack of affective involvement on the part of the caregiver impede the development of affect regulation skills. In the pre-linguistic stage, the child learns to recognize the boundary between self and other through information from the senses and the distinction between self-produced stimuli and those that come from outside (Blanke, 2012). This is made possible by a mechanism that provides for the attenuation of cortical sensory processing (Bays et al., 2006), by which the perception of self-produced stimuli is suppressed. Namely, the brain must predict the sensory feedback of its actions in order to suppress their perception and distinguish them from the perception of external stimuli. This mechanism has also been shown to affect the tactile domain (Boehme et al., 2019): the distinction between externally and self-generated touch forms the basis for the establishment of social bonds. Indeed, touch by others is associated with the activation of areas involved in social cognition, such as the insular cortex and the posterior superior temporal sulcus (Gordon et al., 2013), and it is processed differently depending on whether it is a light touch or a touch that signals affective content (for example, a caress). The latter contributes to the construction of the bodily self. Therefore, the link between somatosensory processing and the formation of the self-concept is evident (Boehme et al., 2019).

The ability to recognize one’s own body and its boundaries as an entity distinct from the environment seems to develop within a few hours of birth (Ronga et al., 2021): by distinguishing whether an auditory or tactile stimulus comes from near or far from one’s own body, the infant is able to develop defensive behaviors and relational mechanisms. Thus, it appears that newborns are already able to make multisensory responses by distinguishing body-related stimuli from stimuli from the environment and that this ability is modulated by the proximity of these stimuli to the body. The near-body space would be represented from the earliest stages of life, predisposing individuals to direct their actions toward a goal, interact with people, respond to threats, and build a coherent bodily representation of themselves (Ronga et al., 2021).

From these recent scientific acquisitions, it is even more evident how physical proximity and social interaction through contact in early attachment relationships, long before the development of language, play a fundamental role in the construction of the self. In fact, in the early stages of development, the search for proximity and the “exchange of safety signals” depends on the movements of the body regulated by the most primitive neural pathways. However, for the sake of completeness, the influence of the individual’s innate/inherited characteristics on hyperreactivity or over-control dynamics must also be considered. In fact, there is considerable evidence that anxious features, emotional dysregulation and impulsivity associated to alterations of reward system also occur in ED (Harrison et al., 2010; Wierenga et al., 2014).

The authors of this article hypothesize that, from the first exchanges between caregiver and newborn already, as well as from the first action patterns and physical contacts, a basis can be created for a functioning that will lead to the development of an ED. We can think that an intrusive and controlling caregiver will use a different touch than a caregiver tuned to the needs of the child, repeatedly invading their proximity space, creating confusion in the coding of signals coming from near and far and compromising the child’s ability to differentiate themselves from the other. If already in the first hours of life, the child is able to distinguish an affective touch from an accidental or non-affective touch, early interactions based on a contact aimed at almost exclusively accentuating the material care of the body could contribute to the construction of a bodily self-representation based on exteriority and esthetics above all as sources of one’s personal value. During development, the body becomes the place where the individual realizes and manifests a personal failure or success. Moreover, the caregiver’s obsessive attention to the child’s body image through the evaluation of shape, weight, size, and musculature can disrupt these processes of delineation between self and others and lead to an overdifferentiation of one’s body, which even becomes an object that can be manipulated. Legrand and Briend (2015) emphasize that in anorexia and the associated manipulation of eating behavior, the transformation of the body is functional to influence relationships with others in order to gain recognition. In this case, the person seeks autonomy but also the recognition of others by showing hunger for the other, not for who the other is as such.

As the theory of embodied cognition (Meteyard et al., 2012) explains, there is indeed a functional unity between the sensory-motor level and the cognitive/perceptual processes. Information processing may be influenced by, modified by, or completely dependent on bodily experiences. In EDs, the person’s behaviors tending to focus on the external features of the body (such as height, weight, and muscles) become tools to communicate the internal state as well as the difficulties in the interactions with the environment.

Since body representations in the early stages of life are the most important tools for generating meaning, organizing experiences, and shaping social identity, we can ask what role bodily interactions and the formation of sensorimotor and proprioceptive patterns play in the development of EDs. Bonev and Matanova (2021) highlight how the lack of attunement and traumatic experiences later affect the ability to think symbolically. The reduction in the ability to know and see oneself as an independent agent can lead to the use of the body to express what cannot be represented.

According to some authors, EDs can be considered as the result of damage to the ability to update a negative representation of the body contained in autobiographical memory with sensorimotor data and current proprioceptive data (Riva, 2014; Fuchs, 2021). According to this view, in patients with ED the representation of the body (“objectified body” or “body-as-object”) could be blocked, unable to contrast with egocentric representations and proprioceptive information. In anorexia, the emphasis on the sole dimension of the body-as-object, the body-for-others, to the detriment of the body-as-subject (in the awareness of the physical and emotional sensations experienced), leads to a reification of one’s individuality with the consequent search for control and self-observation over the external image (Fuchs, 2021). The shame experiences present in autobiographical memory would influence beliefs about oneself and body image, causing a distortion of attention to one’s body. The shame experiences are recalled from the perspective of an external observer, which is associated with an inhibition of insula activity and real-time processing of body experiences. Blocking an allocentric perspective of self and body and reducing the perspective of the embodied self may contribute to a lack of awareness of interoceptive cues (Riva, 2014).

Such non-integrated body states are perceived as a source of dissatisfaction and no longer represent a source of awareness of self. In the typical binge eating episodes of BED and BN, poor monitoring of mental states and a confused understanding of the body’s signals leads to feelings of anxiety, shame, emptiness, and helplessness being misperceived as signals of hunger. On the other hand, a “false caress” exerted by a frightened/anxious caregiver is configured in a “neuroperception” that generates alarm and does not allow to deactivate the defense system in safe conditions or, on the contrary, to activate a defensive behavior in dangerous situations (Porges, 2011).

The repetitive patterns of interaction between caregiver and child, remembered through implicit and procedural memories, influence the formation of memories, and the development of representational processes; the narrative processes, emerging during the first years of life, help to give these representations a sense of continuity over time and to create generalizations, mental models of relationships and basic cognitive structures with which to interact with others (Siegel, 1999). High levels of affective attunement, maternal sensitivity, and responsiveness allow the child, from birth, both to maintain an optimal balance between closeness and exploration behaviors and to profit from mirroring games and conversations in order to formulate inferences about states their own and other people’s mentalities as well as to attribute meanings to the resulting behaviors (Legerstee, 2005).

Thanks to the acquisition of a more articulated language and a more mature ability to represent reality, the child develops a self that integrates memories at a still semantic level; then, the narrative self emerges, through which the child is able to organize past and future experiences into a unitary life story. Specific episodes and social representations of one’s own culture are then integrated (Nelson and Fivush, 2004).

The explicit processes of memory play an important role in contextualizing experiences in time and space; the use of language in communication patterns shapes one’s conscious experience of oneself and the way one relates to others. This helps to determine the “Life themes” in the narratives (Di Fini et al., 2013; Veglia, 2013; Veglia and Di Fini, 2017; Di Fini and Veglia, 2019) around which the elements of implicit memory are integrated. This extends to the creation of narrative processes that allow us to imagine and recall experiences in the form of stories, creating a narrative memory (Siegel, 2014). By considering the connection between somatic memory and narrative memory as a site of identity construction, we can propose a reflection on the construction of the self by people with ED, in light of the narrative function of the body and how it relates to their own bodily boundaries. Food interactions are one such area of a person’s daily life that renegotiates body image and thus influences body boundaries. Thus, food can constitute one of the communication channels of attachment relationships within food interactions (Johnson and Connors, 1987). Non-verbal exchange and interaction leave neurophysiological and representational traces that will serve as a model, organizing the child’s subsequent experiences (Siegel, 2001).

## Attachment and Eating Disorders: From Controlling Others To Self-Regulation

Studies that have examined the relationship between attachment relationships and EDs are numerous and do not always agree in their findings. Empirical studies report a high prevalence of attachment insecurity in samples with EDs, between 70 and 100% (Ramacciotti et al., 2001; Ringer and Crittenden, 2007; Lunn et al., 2012), compared to control groups, both by self-report and by the Adult Attachment Interview (AAI; George et al., 1996; Kuipers and Bekker, 2012; Caglar-Nazali et al., 2014). In several studies of patients diagnosed with AN, BN, and BED, a higher incidence of an avoidant attachment style compared to an anxious one was found (Ramacciotti et al., 2001; Latzer et al., 2002; Barone and Guiducci, 2009). However, there are not many studies that have investigated this prevalence by comparing the different attachment styles or states of mind (SoMs), and the results seem to be contradictory. While some authors report a prevalence of a dismissing SoM, others report a prevalence of a preoccupied SoM, especially when the disorder is accompanied by depression (DeKlyen and Greenberg, 2016). Candelori and Ciocca (1998) found a prevalence of dismissing SoMs in patients with restrictive AN (58%), whereas preoccupied SoM were prevalent in patients with purging AN (50%) and BN (67%), indicating a relationship between dismissive attachment and food restriction versus food elimination and preoccupied attachment. However, the findings of Zachrisson and Kulbotten (2006) and Ward et al. (2001) did not show a specific relationship between the type of anorexia (restrictive vs. laxative) and SoMs. Instead, other authors found a prevalence of a preoccupied SoM (40%) in people diagnosed with AN, whereas in those diagnosed with BN (50%) and BED (70%) the predominant SoM was dismissing (Barone and Guiducci, 2009). Numerous studies have also highlighted the presence of a high incidence of negative and traumatic experiences with unresolved and unsafe SoM in the samples with EDs, underlining that early relational trauma is also a predictor in these psychopathological disorders (Fonagy et al., 1996; Ward et al., 2001; Ringer and Crittenden, 2007; Tasca et al., 2013).

Currently, research does not allow us to take a definitive position, and there is disagreement about the relative prevalence of each attachment style in the ED population (Tasca and Balfour, 2014), as well as the relationship between attachment style and specific ED diagnosis (Kuipers and Bekker, 2012; Tasca, 2019). Therefore, attachment insecurity appears to be a transdiagnostic risk factor that increases vulnerability to ED onset, but only partially explains it. In contrast, other, more specific factors would influence the specific nature of EDs (Kuipers and Bekker, 2012; Tasca, 2019). The change of these factors or the influence of negative, traumatic or relational experiences could lead to a switch between different diagnostic triggers (Rø et al., 2005; Vrabel et al., 2008).

Several authors have also sought to understand the role that attachment insecurity plays in the development and maintenance of EDs. It is well known that effective attunement by the attachment figure does not always occur, leading to crises or “breaks” in attachment. In secure attachment relationships, after the lack of attunement with the child, the parent is able to regulate the child’s affective state in a psychobiologically attuned manner, focusing not only on the child’s manifest behaviors but also on the child’s internal states (Tronick, 1989; Beebe and Lachmann, 1994; Schore, 1994). In order to perform her own regulatory function and mirror the child’s affective state, the caregiver monitors herself, separates herself from the child, and self-regulates by managing this tension so as not to be overwhelmed by it (Krystal, 1978; Fonagy et al., 1996). The parent supports the child in enduring the strongest emotional tensions by intervening just enough to comfort the child and thus modulate communication so that the emotions do not become unbearable and unmanageable. Thus, in the first stage of life, the experience of disturbing affect is externally regulated by an attachment figure who is attuned to and involved in a mechanism of reciprocal emotional communication (Stern, 1985; Beebe and Lachmann, 1988).

A parent who is able to reflect on the child’s mental life, see the mind beyond behavior and respect the existence of their own internal subjective world is fundamental to the development of secure attachment (Slade et al., 2005). Within the attachment relationship, the infant’s immature brain manages to coordinate its activities through the brain processes of the parent (Hofer, 2006). The ability to connect and relate to others is therefore crucial for the maturation of those neural circuits that mediate the capacity for self-regulation, mentalizing, and reflective functioning (Schore, 2001). Fonagy and Target (2001) use the concept of reflexive function to refer to the mother’s mental capacity to think and mentally represent her child’s mental states, to attach subjective and intersubjective meanings to them, and to enable them to internalize this function, which is useful for reading their internal experience. When the mother acknowledges the child’s mental states, the child recognizes that she has the ability to influence reality herself and develops a sense of agency.

As Tronick et al. (1998) points out, isolated moments of nonattunement in the dyadic relationship are normal and do not in themselves have a negative impact on development as long as the individual can still experience repair and attunement. However, when frequent moments of resonance and affective attunement are absent during the first 3 years of a child’s life, we may observe diminished development of emotional regulatory functions and impaired *reflective functioning* (Fonagy and Target, 2001). In these cases, the child’s intentionality tends not to be reflected by the caregiver, leading to difficulties in the maturation of the right hemisphere areas involved in affect regulation. It is likely that high levels of arousal, prolonged, and unregulated, contribute to inhibiting the functioning of the frontal areas of the brain that normally underlie mentalization (Phelps and LeDoux, 2005). The chaotic and destabilizing emotional activation, combined with the loss of hope that one will receive reassurance and co-regulation from the other, results in the continuity of the self being undermined (Bromberg, 1998), with the result that metacognitive skills, such as mentalizing and emotion regulation break down.

In reports of parenting styles of people with EDs, fathers are most often described as dismissive and emotionally unavailable, while mothers often seem to be perfectionistic, controlling, intrusive, or overprotective. Parents tend to inhibit behaviors aimed at their children’s autonomy and independence, resulting in the gradual development of feelings of rejection and inadequacy (Cole-Detke and Kobak, 1996). Often in the reconstruction of the history of patients with EDs, through episodes reported by caregivers or by the patients themselves, it is observed how the family context of growth was characterized by situations of confusion or ambiguity. In these contexts, food can be used as an instrument of regulation and comfort. For example, a worried and anxious caregiver, activated by their baby’s crying, can use food to reduce their alarm. A caregiver who is not tuned with emotionality may interpret crying as a signal of a material and practical need of nourishment for hunger, thus not reflecting the emotional dysregulation underlying that signal, but diminishing it. From many narratives of obese patients or patients with a BED, memories related to the care and comfort offered by alternative attachment figures in accompaniment to or through food emerge. In these cases, the patients’ narrative memory about safety, emotional regulation, and loving kindness seems to be associated with the somatic memory of comfort through a special food. However, the perpetration of an uncontrolled mode of eating would seem to be connected to the lack of an emotional awareness about the meaning of that gesture and its compulsive search or to the underlying need for comfort and kindness (Verschueren et al., 2021).

In these scenarios, communication is strongly connected to the feeding. In the lack of attunement, the opportunity for the child to acquire the ability to recognize, disambiguate, and differentiate the connotation of their own needs, necessities, and discomforts underlying a moment of activation, such as crying, is lost. In addition, the constant violation of personal boundaries, common in ED families, by intrusive parents who tend not to delineate and substitute for their child, leads them to be confused about themselves, others, and self-definition (Selvini Palazzoli, 1988). These repeated failures are the basis for attachment insecurity and the resulting lack of emotion regulation (Liotti, 2001). In many cases, this insecurity takes the form of alexithymia, characterized by poor emotional awareness, with an inability to recognize the arousing effect on somatic elements and an operative cognitive style, a condition that often underlies the development of psychopathological situations with somatic implications (Taylor et al., 2006b). Alexithymia appears to play a mediating role between the presence of attachment insecurity and body dissatisfaction (Keating et al., 2013). The latter is an important factor in predisposing to EDs (Troisi et al., 2006; Abbate-Daga et al., 2010). It has also been associated with two aspects commonly found in patients with AN and BN, namely, the need for approval and the fear of rejection, both of which are relevant factors in attachment anxiety. Greater attachment anxiety is associated with greater symptom severity and poorer therapeutic outcomes (Illing et al., 2010), and it appears that it is emotional dysregulation that mediates the impact on ED symptoms.

These studies support the perspective that attachment insecurity, along with a lack of emotional regulation skills, plays a role in the development of and resistance to change in EDs. When the experiences of emotional neglect occur in the relationship with the caregiver, mental closeness becomes painful and the natural need for closeness tends to be expressed at the physical level, for example, through excessive attention to body-oriented material care and feeding. Indeed, there is a high prevalence of neglect, particularly emotional neglect, in the lived experience of people with ED compared to the general population (Pignatelli et al., 2017). Emotional neglect, included among the Adverse Childhood Experiences (ACE, Felitti et al., 1998; Van der Kolk, 2014), affects the physical unhappiness (Grenon et al., 2016) of people with ED, where emotions can be read and expressed primarily through the body, while mental abilities are impaired, which does not allow for adequate mental representational capacity. The inability to think through representations that may contain an experience means that they are experienced as concrete and tangible facts through the body (Skårderud, 2007). In EDs, symptoms are concrete (Buhl, 2002), physical, and described by body image, shape, and weight. The body triggers emotional experiences, just as actions trigger physical sensations; therefore, abstract meanings are absent in favor of concrete experience (Skårderud, 2007). This process can be explained by a deficient reflective function leading to immature functioning, such as “mental equivalence,” where mental states are perceived as extracts of objective reality (which is certainly true) rather than mental representations (Bateman and Fonagy, 2004), indicating weakened development of symbolic ability (Enckell, 2002).

Research that has looked at mentalization has confirmed that people with EDs lack this competence (Kuipers and Bekker, 2012) and has found that this is a specific factor in the diagnosis of certain EDs, particularly AN (Fonagy et al., 1996; Rothschild-Yakar et al., 2010). At the same time, inadequately controlled affective regulation has been shown to be associated with eliminative eating behaviors (Candelori and Ciocca, 1998) and overly controlled with restrictive eating behaviors (Stice and Fairburn, 2003).

In addition to early attachment relationships and the development of emotion regulation skills, consideration must also be given to the role of personality and innate/inherited traits for which ED is a way of reenacting or communicating these issues. In fact, also considering personality traits allows for a better understanding of the heterogeneity of symptom profiles, the strong or critical points for treatment adherence, and the different treatment prognoses (Farstad et al., 2016). In patients with borderline personality traits, emotional dysregulation was found to be a negative predictor of treatment outcomes (Muzi et al., 2021). In addition to emotional dysregulation, impulsivity also plays an important role in patients with ED, especially in those with BED and BN. It contributes to greater levels of psychiatric and eating disorder symptoms (Favaro et al., 2004) and greater symptom residuals at the end of treatment (Martinez and Craighead, 2015). Other important personality traits in ED phenomenology include perfectionism and obsessive–compulsive traits. The former is associated with a longer clinical course of suffering, while high levels of obsessions about food, weight, and body shape are associated with higher severity of disordered eating behaviors and prognosis. Avoidance of experiences, characterized by aversion to internal states, is also prominent in patients diagnosed with eating disorders. In this case, eating symptoms would allow them to cope with the negative or too intense affects they are trying to avoid (Martinez and Craighead, 2015).

Another characteristic feature of EDs, and in particular of AN, is cognitive rigidity, which is important for the development and maintenance of the disorder (Tenconi et al., 2010).

**Figure 1:**
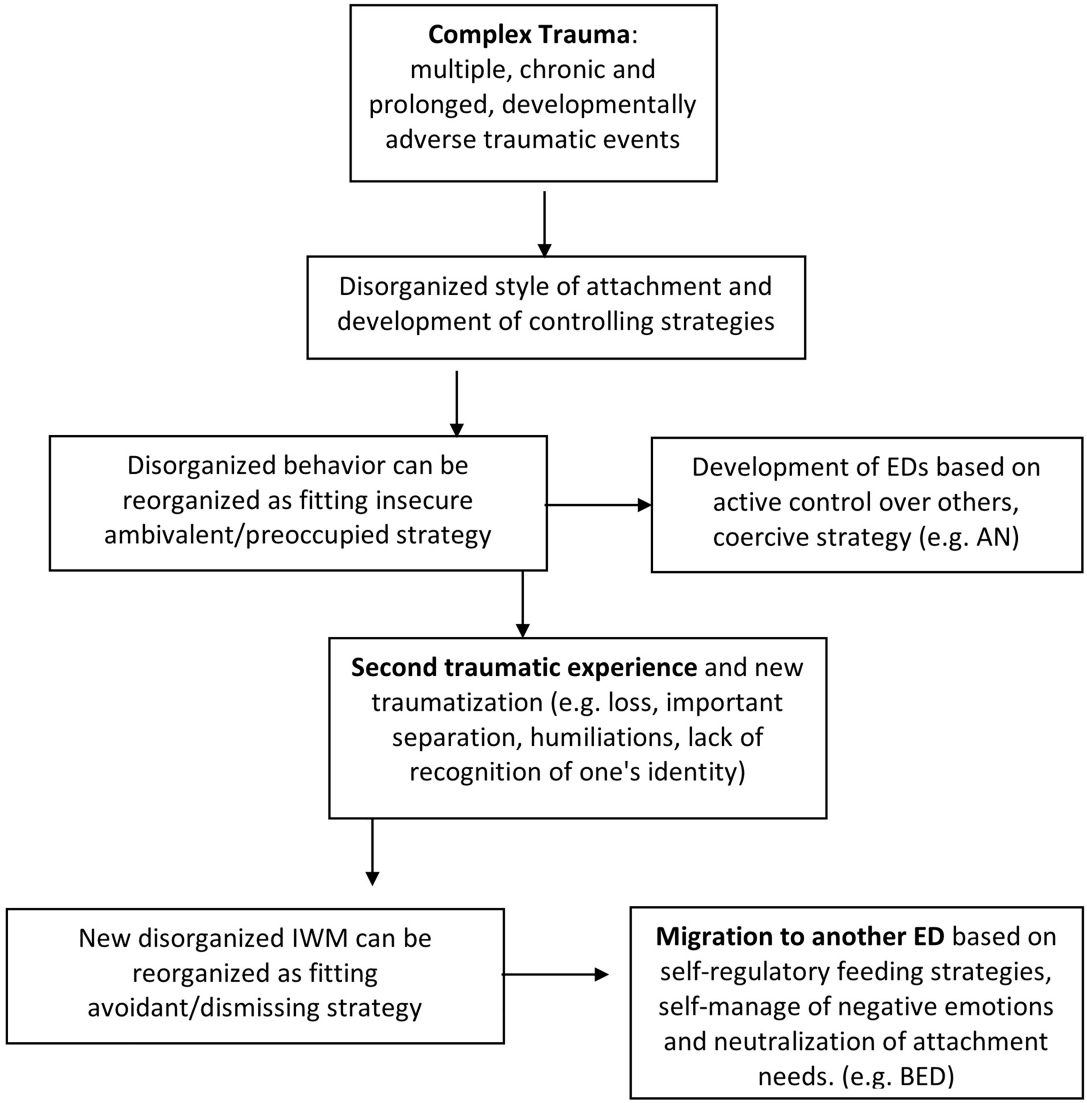
The diagram summarizes the proposed hypotheses about the transition from one set of symptoms to another in the histories characterized by complex trauma, taking into account the effects of traumatization and the consequent reorganization of attachment and regulation strategies.

Years of clinical observations in the field of ED have led the authors of this article to note a particular phenomenon in patients with ED and a history marked by adverse childhood experiences, which is simultaneously associated with regulatory strategies and symptoms. As found in the literature (Fairburn and Harrison, 2003), a change in ED symptomatology or a transition from one dysfunctional eating behavior to another was noted in many cases during the course of treatment. At the same time, a shift from one emotional regulation strategy to another was observed over time. In order to try to provide an explanation for these phenomena observed in clinical practice, the authors propose an explanation of the possible mechanism that takes into account the effects of traumatization, the changes in behavioral strategies, and the transition to different dysfunctional behaviors of eating and EDs. The mechanism schematized in Figure 1 considers cases of ED, in which the process of traumatization combined with a disorganized attachment following adverse relational experiences may have played a role in the development and maintenance of symptoms.

Starting from developmental trauma (for example, due to repeated experiences of emotional neglect in childhood, common in the narratives of patients with EDs; Pignatelli et al., 2017) and the resulting disorganization of the internal working model (IWM), the attachment system seems to reorganize around the sixth year of life. Strategies defined as “controlling” are developed (Lyons-Ruth and Jacobvitz, 1999; Liotti, 2004), which, in order to maintain closeness to the caregiver, activate the rank motivational system (punitive-dominant strategy) or the caregiving strategy instead of the attachment system. Furthermore, following the theories of Crittenden (1992), the authors of this article hypothesize that in EDs, the attachment style reconfigures and reorganizes itself in an insecure style with anxious-ambivalent or mixed tendencies, belonging to the A/C defended/coercive classification. This reorganization would include a relational modality that implies the control of the other and the management of power over the other, typical of the relational exchanges of EDs, in particular AN. Symptomatology gives the individual power and control over others since, thanks to this, the relational balance of the family is modified (Selvini Palazzoli, 1988). At a semantic and narrative level, power is a central theme in families whose members have EDs (Bruch, 1973; Castiglioni et al., 2013; Ugazio, 2013). The central emotions are those of pride if superiority is recognized to the conversational partner, and shame if, on the other hand, a defeat is perceived (Ugazio, 2013). The polarities on which these people move are winner-loser and strong-will surrender, which is subordinate to the former for a means-ends relationship, as the person is a winner if they are also strong-willed and is a loser if they are passive (Ugazio, 2013). The content of this semantics is purely relational as the people who use it can define themselves as winners or losers only with respect to others. Perceiving oneself as a winner or a loser is not individual, but is only allowed in a relationship, after a comparison based on rank differences (Ugazio, 2013).

At the same time, the anxious-ambivalent attachment style in the caregiver is accompanied by a poor capacity for self-regulation in the eating behaviors of the child; this regulation seems to be mediated by a controlling and persuasive attitude by the caregiver. Practices linked to a restriction of certain foods, bans, and punishments or positive reinforcements through particular foods would lead the child to no longer respond to their own sense of hunger or satiety, but rather to react to the pressing requests of the caregiver in exchange for approval and comfort. The relationship with an inconstant, unpredictable and intrusive caregiver in their care behaviors leads the child to develop an unreliable sense of self in decoding internal states, with a consequent sense of personal vagueness and indefiniteness (Guidano, 1988; Anderson et al., 2012; De Campora et al., 2016). With adolescence, the food symptom signals troubles through the transformations of the body which, in an ambivalent way, requires attention and care on the one hand and rejects them on the other.

However, it is interesting to note that in clinical work the transition over time toward an avoidant-type style underlying EDs is increasingly observed. If the ambivalent attachment style involves the need to stay in a relationship by hyperactivating the attachment system, the avoidant style allows one to avoid the relationship that has not been regulative and to aim for self-regulation (Schore, 2003). A strategy based on “do-it-yourself,” autonomy, and relational withdrawal as a way of regulating affect, rather than hetero-regulation (in which the individual uses others to regulate him- or herself) or co-regulation, is associated with some parent feeding styles (Hughes et al., 2011; De Campora et al., 2016).

The migration from a set of symptoms to another one could be explained by the arrival, at a certain point of life, of a second traumatizing factor (for example, a bereavement, a significant separation or the disavowal, and humiliation of one’s identity by others). This would imply a new phase of disorganization of the system and a new fluidity of the IWM which would be followed by a second reorganization. In fact, the research reports how, in response to some vulnerability factors and particularly stressful life events, the SoM can change (security levels are subject to fluctuation; Mikulincer and Shaver, 2016). This phenomenon appears to be more likely for individuals who have had early traumatic experiences and who have developed more unstable and nuanced models of self and others (Davila and Cobb, 2003). The authors of this article therefore hypothesize that, in correspondence with a new opportunity for reorganization, the system may migrate toward an avoidant style and toward the development of a self-regulatory feeding strategy. This is in line with the promotion of deactivating and distancing strategies for managing distress (Powell et al., 2017). Where the controlling strategy collapsed, for example, a strategy of a punitive type, it would move to a strategy based on self-control, accompanied by a social withdrawal and a mode of nutrition aimed at self-consolation (typical of BED and obesity). Having been humiliated, or thinking oneself capable of being humiliated, leads to experiencing shame as a predominant emotion in the EDs. During binges, the sufferer tries to regulate the feeling of shame, but it is exacerbated by the binge itself and the inability to control one’s eating. Sometimes the disorder then withdraws itself so as not to be disturbed in its self-regulation, rather than seeking it out, while neutralizing attachment needs.

By withdrawing and using avoidance strategies to reduce criticism from others, there are fewer opportunities to have sufficient communicative exchanges with the other, thus also reducing the possibility of reparative and corrective experiences. In adolescence, even with peers, inadequate interaction and a modality based on forced autonomy and withdrawal leads to the individual not developing the measure of their own acceptability/non-acceptability. Therefore, avoidance feeds the feeling of social inadequacy in a dysfunctional interpersonal circle (Carcione et al., 2016). According to the theoretical framework of attachment (Bowlby, 1973), the experience of inadequacy and the sense of loss of control, characteristic of EDs, emerge in the child when the caregiver, through the relationship, conveys to the child that the child lacks amiability and adequate ability to cope with life. In these cases, the development of an avoidant attachment style seems to be aimed at the possibility of diverting attention from the inner emotional distress. In an ED, such an attempt would find a way of realizing one’s control oriented approach toward an external and tangible objective that is easier to manage (DeKlyen and Greenberg, 2016). In summary, if the ED and the body initially attract the attention of the family, signaling a struggle for self-esteem, autonomy and separation, and a hunger for recognition (Fuchs, 2021), they later become the place to self-manage one’s negative emotions and neutralize attachment needs.

The underlying process thus hypothesized could therefore reconcile the conflicting results of the studies that in the samples of subjects with EDs. It could also highlight the presence of separation anxiety, fear of abandonment and control strategies over others in relationships typical of preoccupied SoM on the one hand (Barone and Guiducci, 2009) as well as a predominance of unsolved and dismissing SoM on the other (Zachrisson and Skårderud, 2010; Delvecchio et al., 2014). Therefore, from the perspective of the complexity of EDs, we can consider multiple functioning in different periods of life of the same individual, not just multiple diagnoses.

## The Semantic Re-Traumatization

Clinical experience in the field of psychotraumatology has led to the identification of another type of trauma that occurs when an individual’s sense of self is threatened and develops in interpersonal relationships. In such cases, we can speak of semantic trauma (Veglia, 1999, 2013), since the traumatization process takes place through the path of attribution and exchange of meanings, primarily involving third-level motivational systems (epistemic motivational systems; Liotti and Monticelli, 2008) and thus the neural networks of the neocortex (Panksepp, 2004). This type of trauma is associated with disintegration, fragmentation, and loss of the sense of self built in relationships on a semantic and narrative level. In the context of ED, the process of traumatization, which is primarily concerned with personal worth, the ability to be oneself, and the sense of kindness, as well as the memory of humiliations suffered during development, can be triggered not only by the words received, but also by extra-linguistic experiences in the form of looks, gestures, etc.

Starting from a bottom-up traumatization, a dysregulation of the memory-consciousness system develops and could recursively expose the subject to semantic re-traumatization. The feeling of dysregulation is not organizational from a neurovegetative point of view (dysregulation of the tolerance window) nor from an interpersonal point of view (Interpersonal Motivational Systems – IMS are activated in an incongruent way or, in the case of attachment trauma, through controlling strategies), thus developing a feeling of helplessness. After traumatic experiences, the left brain, with or without words, must inevitably make sense of what is happening (Gazzaniga, 2006, 2011) and is forced to form an image of itself as traumatized. We can say that the brain “tries again” in a cortical way to understand what happened: from this operation emerge the meanings and “negative” or “pathogenic” beliefs about oneself and the world. The negative beliefs thus stem from the reactivation of the cortex immediately after the trauma, which tries to reorganize itself to ensure that what happened is congruent with the other possible meanings it can form, and to translate “the extraordinary” into the canonical and intelligible (Bruner, 2000). For example, the threat of humiliation no longer comes from the outside, but from a constant self-deprecating attitude. This way of thinking about oneself becomes the source of re-traumatization (e.g., irrefutable memory of being bad and unworthy) as a consequence of post-traumatic “adaptation” (see the concept of “accommodation” in Piaget, 1952 and “self-deception” in Guidano, 1988).

Semantic memory and narrative memory are the result of reorganizing the meaning of an event that was originally meaningless. In trying to make sense of it, the person becomes retraumatized to the point where the mere activation of this negative belief about himself is enough to restart his alarm system. When the person is in a critical situation that triggers severe stress, he or she does not necessarily activate the original traumatic memory (e.g., direct humiliation in the past), but rather the self-representation available at that moment that was developed to explain the traumatic experience. This self-representation in EDs manifests primarily in beliefs, such as “You are fat,” “You have no control,” “You are not worth anything,” and “You are incapable of not eating.” These reactivate the alarm system in a dysregulated manner and cause the individual to re-trigger the action patterns developed during development for self-regulation, such as avoidance behaviors or fight-flight patterns.

According to Foa et al. (1991), what makes memories “traumatic” is a combination of the original experience of the trauma being stored as it actually happened and the subsequent visual reproduction and linguistic representation of the memory. The representations of the event change as they are experienced along with a variety of cognitive and affective associations that give meaning to the traumatic event in a continuous process. Eventually, the affective networks activated by the memory of the trauma may come to dominate the neural network when later memories of the event occur. Lanius et al. (2011) also consider the impact of psychological trauma on the sense of self and disturbances in self-referential processing, as they note that post-traumatic cognitions associated with a sense of shame would form following trauma.

Thus, this mode of functioning goes beyond the notion of traumatization as activation caused solely by triggers related to unresolved experiences, and envisions top-down traumatization through beliefs and narratives about oneself based on lack of kindness, devaluation, and an inaccessible truth regarding what others think of oneself in limited spaces.

According to the theories of Tronick et al. (1998), newborns, as open dynamic systems, need to constantly gather information to increase their complexity and coherence. They satisfy this need by converting non-verbal meaning into a “biopsychosocial state of consciousness” that shapes their ongoing engagement with the world. Psychological problems in children arise when the meanings ascribed to the situation selectively limit their subsequent relationship to the world and thus the long-term growth of their state of consciousness. When chronic and repeated, these altered meanings can impair development and increase vulnerability to pathological outcomes. This perspective views trauma as interrupting and distorting the creation and selection of meanings, in no way neglecting the dysregulatory, disorganizing, and dissociative effects of traumatic events. Trauma would thus limit the expansion of complexity in open dyadic systems by interfering with the processes of defining new meanings. Moreover, the toxic effects of early relational trauma on young children are amplified over time because these effects distort and exclude other typical meaning attribution processes that underlie positive developmental outcomes (Tronick and Beeghly, 2011).

In the stories of patients with ED, we often find experiences of constant confrontation with brothers/sisters, who were more valued and respected by caregivers, or external models (thin-ideal internalization), disqualifying, and disabling relational experiences, such as experiences of recognition that are mainly associated with achieving outstanding results or accomplishment (Taylor et al., 2006a; Setiadi and Risnawaty, 2021). When the parental figures do not allow the individual to be themselves, to express and build their sense of self, to realize their potential and freely make sense of their own existence, a painful and torn wound is created. In fact, in the development of the IWM, which, at the level of internal representations, is configured as a prerequisite for the construction of the self, the constraint of maintaining coherence and continuity in one’s self may conflict with the constraint of maintaining closeness and relationship with the caregiver. When the sense of self enters a crisis in relation to critical narrative themes, there is a sense of not being adequate in interactions and having to give up an authentic part of oneself in order to maintain a sense of security. This is followed by a threat to the narrative development of the themes of personal worth and likability (Veglia and Di Fini, 2017; Di Fini and Veglia, 2019). For example, if the relationship with the caregiver generates alarm, restrictive eating behaviors may be a way to maintain a sense of control over the relationship and a powerful sense of self; however, this creates disapproval from the outside. Conversely, the price of maintaining a positive external evaluation is a sense of helplessness in the relationship and a negative internal representation of oneself. At this point, the individual finds himself in the paradoxical situation of having to choose between losing a sense of security in the relationship while maintaining their sense of self, or, conversely, losing their sense of self while maintaining a stable attachment. This dilemma leads to a sense of helplessness because he/she cannot be both good and bad, lovable and despicable, and victim and guilty (top-down semantic traumatization). The failure of the narrative, the lack of meaning and the contradictory nature of the meanings create a new, protracted alarm and recursively retraumatize the individual (reactivation of the semantic traumatization cycle from bottom to top). The result is therefore a fragmentation of narrative coherence and continuity, leading to a dysregulation of IMS activation and arousal.

For example, if perfectionism, as a characteristic regulatory strategy of some EDs, aims, on the one hand, at obtaining affirmations from others in support of one’s own worth, on the other hand, it constantly exposes the individual to an unbearable sense of shame and emergent unworthiness because he or she considers him- or herself inferior and uninteresting. In the disorganization of attachment that characterizes traumatic relational contexts, the state of fear without solution can lead to a deficit in the integrative functions of consciousness and a greater susceptibility to dissociative reactions and a fragmentation of the self in response to traumatic stressors during development. Problems with self-esteem, a deep sense of shame and inferiority, and diversity act as mediating factors that increase the likelihood of using food to cope with the disturbed self-image. Prolonged and repeated traumatic shame experiences tend to lead to fragmentation of the self, which is the extreme dissociative form. At a high level, chronic shame does not necessarily require the real presence of another person, but merely the internal image of how another person might judge one. Chronic shame, then, is fueled by humiliating internal images that may stem from past experiences without necessarily reflecting the current situation.

## Proposal For a Multi-Access Model of Psychotherapy

In recent decades, the methodological approach on which cognitive behavioral therapy is based has spawned several conceptual frameworks and numerous scientifically based intervention methods. However, these are often limited to the part of the brain or to the mental functions that each individual author or research group or school of thought has focused its attention on during a particular historical period. The goal of the various explanatory models of the mind or brain is to identify, describe, demonstrate, and formalize the mechanism that explains the physiology and pathology of mental functions and enables the creation of the most effective intervention plan possible. However, this very often conflicts with other explanatory models. These methods have been derived from reference theories of great epistemic consistency, such as evolutionism, constructivism, constructionism, developmental psychology, the metacognitive approach, and the bottom-up approach. Each of these areas can be studied as a pathway for activating/deactivating the brain/mind system or as a predominant processing center for the organism’s internal and external signals or for regulating actions, mental states, and interpersonal positions.

Clinical observation and the results of neurobiological research, albeit preliminary, illustrate the ongoing reciprocal and overlapping influence of every activity in every area of the brain and in every domain of mental experience, both *via* interhemispheric conduction and *via* top-down/bottom-up pathways. The question of whether a strong and sudden threat signal stimulates flight rather than thought, or whether a persecutory ideology produces a strong sense of threat *via* the cortical pathway that causes one to fight or flight, is the result of conceptualization by opposites that fails to capture the complexity of the situation. Currently, cognitive and behavioral psychotherapy consider different approaches to orienting the patient in therapy, configured as predominantly declarative (based on cognitive, logical, linguistic, and narrative interventions), predominantly affective and relational (based on interpersonal, metacognitive, and emotionally motivational interventions), or predominantly procedural (based on behavioral, sensory, motor, and neuroregulatory interventions). Although the effectiveness of each of these approaches has been demonstrated, there is no conclusive evidence that one is more effective than the other. What distinguishes them from one another is their use in the creation, planning, implementation, and ongoing reformulation of the plan of care. Each approach is appropriate to functionally support parts of the treatment plan and to keep the links between reflection and clinical action consistent. Thus, the choice between different approaches, techniques, and content should depend on the patient’s history and personal processing modalities of critical experiences (agency, mastery, and resilience) rather than on nosographic diagnosis or therapist preferences. It should relate to the gene expression of their DNA, which has been favored by the physical and relational context in which they have lived, to the way life has shaped their brain, to what they have learned from the experiences, and to their unique and unrepeatable way of consciously using these experiences and communicating them to themselves and others as an expression of their autobiographical consciousness.

The Multiple Access Psychotherapy (MAP; Veglia et al., 2019) model refers to the choice of method, starting from the self-referential way in which the individual tends to build the idea of themselves and themselves in the world. Some do this through verbal and linguistic narratives or through schemas of action, while others do so through schemas of relationship. These are different modalities of semantic attribution that are probably biologically predetermined, as a request from the brain structure to work more easily in certain modalities. These are also the most likely access pathways for finding the specific locus of the patient’s suffering. “Multi-access” refers to the indication for the clinician to go where the patient would pass in order to report their suffering, to successively uncover and explore other access routes, to enrich the work and increase the flexibility, integration and degrees of freedom of the system, and to offer more possibilities to the individual. The clinical work begins with the current suffering expressed according to the personal modalities of each patient. The clinician records it, reads it and describes it together with the patient in order to understand how it manifests in the form of cognitive, emotional, somatic, behavioral, and relational deficits or dysregulations. To work in this direction, the clinician uses the tools of critical sequence analysis as they relate to functional analysis.

Clarifying the experience of pain within the context of a therapeutic alliance reduces the sense of paralysis, disorientation, confusion, fragmentation, and anxiety associated with the inability to understand one’s suffering, and therefore reduces the sense of helplessness that comes from trying to respond. Within the therapeutic session, the development of new emotion regulation skills also takes place through the construction and repair of the alliance, each time trying to maintain a cooperative interaction (Liotti and Monticelli, 2014). Often, some of the suffering remains incomprehensible because it does not seem to be associated with any known antecedents. It is the pain that is evoked by memory, often by unintegrated memories that can suddenly burst into the present, and it is activated by triggers that are difficult to identify. The study of life histories allows us to trace maladaptive learning, punctual traumatic events, or repeated and complex relational traumas with evocative tools capable of detecting the narrative scripts most likely to be associated with current suffering (Pellegrini and Veglia, 1999). The therapeutic relationship is necessarily involved and the body is not only a place of encounter with the other, but also an instrument of therapy. The most important signs of welcome, willingness to listen, acceptance, loving kindness, and empathic sharing are somatic rather than linguistic. If the clinician’s arousal fluctuates within the tolerance window, the tone of voice, rate of breathing, and willingness to make eye contact are likely to activate the patient’s ventral prosocial vagus system, which acts as a regulator of his own state of alarm more than any reasoning about his misconceptions (Porges, 2011).

On the other hand, the memory of many adverse childhood experiences is compromised by the prevailing brain stem and midbrain activation at the expense of the cortical integration areas activation and, in this case, the bottom-up approach offers a way to reactivate them in safety conditions. Thus, it helps individuals to integrate traumatic memories and finally express and make a cathartic gesture. For some patients, however, the main road to change is the construction of new knowledge, new skills, and alternative views on their problems.

Reducing negative thoughts, using multiple points of view, increasing positive mental representations, acquiring the ability to read interpersonal signals and automating the production of more adequate responses to complex social situations with exercise already offer a feeling of competence and security and improve self-image, arousal regulation, and mood. Reconstructing, rearranging, re-reading and narrating, listening, giving back and sharing are powerful evocative and transformative movements in narrative approaches to knowledge and change. The approach is only partially characterized by the use of the word, since the narrative is the product of the whole person and their story, of the characters they are and have been, of their memories and of the possible selves that they will be.

Thus, we have three words for three ways of accessing memory which show how much of the entire mind is involved in its body in the simple act of recalling an event (giving it a voice): remembrance (memory of the body and of the limbs); affective memory (memory of the heart); and reminiscence (cognitive memory of knowledge).

The literature has highlighted how essential it is to take into account the influence of attachment and the factors connected to it in the development and maintenance of EDs. Different styles have also been observed within the same diagnostic category (Wei et al., 2005), with the suggestion to consider the patient’s specific attachment style in the clinical strategies to be used when planning interventions (Tasca, 2019). For example, it was observed that, for BED patients, the treatments in which an opportunity for experience and emotional expression was experienced promoted and increased the therapeutic alliance and the reflective functioning (Compare et al., 2018; Maxwell et al., 2018). Moreover, literature (Zaitsoff et al., 2015; Graves et al., 2017) shows that, especially in the early stages of therapy, the improvement and stabilization of symptoms is accompanied by an improvement in the quality of the therapeutic alliance, which in turn opens up the possibility of working more deeply. Therefore, a relational context characterized by attunement allows the experience of mirroring and co-regulation, acquiring an alternative way to regulate the arousal and improving metacognitive abilities and their consequent mastery (Fonagy et al., 2002; Schore, 2003). This is even more true in cases where ED symptoms are initially hidden from the patient. Working with the therapist to also recognize the adaptive significance of some eating behaviors will help increase the ability to self-reflect, develop a more integrated sense of self, and find new ways to cope with disturbing feelings (Barth, 2008).

The MAP further orients us to the subjective specificity of the patient. It is not the patient who adapts to the therapeutic model, but the treatment plan that must take shape around their relational modalities of meaning construction, alarm management, and expression, considering the factors related to the development history implied in the maintenance of the ED.

According to this perspective, EDs do not correspond to a diagnosis that involves the application of a specific intervention protocol: the patient is placed at the center and teaches the clinician where to go from the beginning, according to their preferential access, to meet their suffering and to begin to understand it. Taking into account the mechanism schematized in Figure 1, MAP can more easily adapt to changes in regulatory strategies, to the shift to different dysfunctional eating behaviors, to the transition from one relational style to another sometimes observed in ED patients. By considering different accesses and different perspectives on the patient’s suffering, we are able to understand its complexity and its processes over time. This tuning to the patient’s preferences is also oriented according to their preferential access way, allowing a consolidation of the therapeutic alliance. This allows the patient to gain confidence in being accompanied in the exploration of other methods. If a patient were more oriented toward bodily concreteness, with a poor reading of their own internal states, we would avoid refuting with them in a declarative way in the first instance. For example, in the theme of control, if we were faced with a patient withdrawn relationally and oriented toward the acquisition of knowledge and the sharing of meanings, it would be more appropriate to use a more cognitive and narrative approach. Meanwhile, if the patient were oriented to action and to experience bodily, it would be advisable to seek relational engagement through the use of bodily actions and a procedural method.

From a clinical point of view, the authors of this article try to highlight the proposal of an integrated model with multiple access to therapy that takes into account the complexity of patients with ED in which the aspects related to dysregulation, non-integration of the perception of one’s own body and post-traumatic symptoms are associated with a sense of suffering self and a self- retraumatizing narrative. Through a sensorimotor approach, able to assess the somatic capacities of the patient and therefore to elaborate a joint plan to observe the defensive reactions of the body, it is possible to structure alternative somatic regulation strategies instead of those related to nutrition. EDs, especially AN, often arise from an attempt to change the structures and balance of the person seeking change through the body (Skårderud, 2007). The body then becomes the incorporated site of synthesis of what the person is seeking. Therefore, the body in therapy can become a place of self-encounter, renegotiation of boundaries and exploration of what cannot be said or done with words. Awareness of one’s own body through the scanning and self-observation of all somatic signals enables the patient with ED, to “stay with it” without avoiding feelings and without being overwhelmed by painful sensations. As the patient learns to notice his or her own habitual responses and relationship to the limits of his or her body, and learns to accept alternative somatic responses, a sense of greater control over arousal is facilitated. The sense of control and mastery therefore counteracts the feelings of vulnerability, helplessness, and shame characteristic of EDs. By working on the modalities of meaning attribution (based on control mechanisms, co-regulation, or semantic exchange of critical narrative themes), narrative integration, and access to mental and somatic states from which one had become alienated, we can help the patient reconstruct his or her own bodily and narrative identity. Thus, if the patient is able to tell his or her own story in different ways (somatically, interactively, or linguistically), this can lead to a deep integration between his or her brain, body, and connection to others.

## Conclusion

Combining clinical observations with recent findings from the literature, this article proposes a reflection on the functioning of EDs that includes the impact of early relational trauma on emotion regulation strategies, the role of attachment relationships in the development and maintenance of these disorders, the narrative construction of the self and the symptom, and connections with somatic memories. Studies of early interactions and physical contact with attachment figures, as well as the literature on the role of mirroring and emotional attunement in self-construction, even before the emergence of language, may shed light on the mechanisms leading to the development of EDs and its associated body representations.

Studies that have examined attachment relationships in the context of EDs report conflicting data on the prevalence of attachment styles and states of mind in these disorders in general, as well as across EDs specific diagnoses. However, a complex and multicomponent perspective that considers the role of attachment insecurity, mirroring, emotional attunement within early mother–child interactions, the impact of traumatization, and semantic re-traumatization in attempting to maintain a coherent sense of self may offer new insights into the development, maintenance, and also the modifiability of eating disorder symptoms. In line with these considerations, the proposed model MAP focuses on the self-referential modalities through which the individual tends to build the representation of the self and of oneself in the world, adapting to one’s preferred “access route” to cope with suffering.

## Data Availability Statement

The original contributions presented in the study are included in the article/supplementary material, and further inquiries can be directed to the corresponding author/s.

## Author Contributions

FV and RAP contributed to the development of the ideas and hypotheses, provided the theoretical insights and clinical foundations, and contributed to drafting and revising the manuscript. GDF and SF contributed to the search of literature, drafted and revised the paper, and translated the paper in English Language. All authors contributed to the article and approved the submitted version.

## Conflict of Interest

The authors declare that the research was conducted in the absence of any commercial or financial relationships that could be construed as a potential conflict of interest.

## Publisher’s Note

All claims expressed in this article are solely those of the authors and do not necessarily represent those of their affiliated organizations, or those of the publisher, the editors and the reviewers. Any product that may be evaluated in this article, or claim that may be made by its manufacturer, is not guaranteed or endorsed by the publisher.

## References

[ref1] Abbate-Daga G. Gramaglia C. Amianto F. Marzola E. Fassino S. (2010). Attachment insecurity, personality, and body dissatisfaction in eating disorders. J. Nerv. Ment. Dis. 198, 520–524. doi: 10.1097/NMD.0b013e3181e4c6f7, PMID: 20611057

[ref2] Anderson S. E. Gooze R. A. Lemeshow S. Whitaker R. C. (2012). Quality of early maternal-child relationship and risk of adolescent obesity. Pediatrics 129, 132–140. doi: 10.1542/peds.2011-0972, PMID: 22201144 PMC3255468

[ref3] Barone L. Guiducci V. (2009). Mental representations of attachment in eating disorders: A pilot study using the adult attachment interview. Attach Hum. Dev. 11, 405–417. doi: 10.1080/14616730902814770, PMID: 19603303

[ref4] Barth F. D. (2008). Hidden eating disorders: attachment and affect regulation in the therapeutic relationship. Clin. Soc. Work. J. 36, 355–365. doi: 10.1007/s10615-008-0164-2

[ref129] Bateman A. Fonagy P. (2004). Psychotherapy for Borderline Personality Disorder: Mentalization Based Treatment. UK: Oxford University Press.

[ref5] Bays P. M. Flanagan J. R. Wolpert D. M. (2006). Attenuation of self-generated tactile sensations is predictive, not postdictive. PLoS Biol. 4:e28. doi: 10.1371/journal.pbio.0040028, PMID: 16402860 PMC1334241

[ref6] Beebe B. Lachmann F. M. (1988). The contribution of mother-infant mutual influence to the origins of self-and object representations. Psychoanal. Psychol. 5, 305–337. doi: 10.1037/0736-9735.5.4.305

[ref7] Beebe B. Lachmann F. M. (1994). Representation and internalization in infancy: three principles of salience. Psychoanal. Psychol. 11, 127–165. doi: 10.1037/h0079530

[ref8] Blanke O. (2012). Multisensory brain mechanisms of bodily self-consciousness. Nat. Rev. Neurosci. 13, 556–571. doi: 10.1038/nrn3292, PMID: 22805909

[ref9] Boehme R. Hauser S. Gerling G. J. Heilig M. Olausson H. (2019). Distinction of self-produced touch and social touch at cortical and spinal cord levels. Proc. Natl. Acad. Sci. 116, 2290–2299. doi: 10.1073/pnas.1816278116, PMID: 30670645 PMC6369791

[ref10] Bonev N. Matanova V. (2021). Adult attachment representations and body image. Front. Psychol. 12:724329. doi: 10.3389/fpsyg.2021.724329, PMID: 34566806 PMC8461008

[ref11] Bowlby J. (1973). “Attachment and loss: volume II: separation, anxiety and anger.” in Attachment and Loss: Volume II: Separation, Anxiety and Anger (London: The Hogarth press and the institute of psycho-analysis), 1–429.

[ref12] Bromberg P. M. (1998). Standing in the Spaces: Essays on Clinical Process, Trauma, and Dissociation. Analytic Press, Mahwah.

[ref13] Bruch H. (1973). Eating Disorder: Obesity, Anorexia Nervesia and the Person within, Basic Book. New York.

[ref15] Bruner J. S. (2000). La cultura dell’educazione. Nuovi orizzonti per la scuola. Feltrinelli editore.

[ref131] Buhl C. (2002). Eating disorders as manifestations of developmental disorders: language and the capacity for abstract thinking in psychotherapy of eating disorders. Eur. Eating Disord. Rev. 10, 138–145. doi: 10.1002/erv.440

[ref16] Caglar-Nazali H. P. Corfield F. Cardi V. Ambwani S. Leppanen J. Olabintan O., . & Treasure J. (2014). A systematic review and meta-analysis of ‘Systems for Social Processes’ in eating disorders. Neurosci. Biobehav. Rev., 42, 55–92. doi: 10.1016/j.neubiorev.2013.12.002, PMID: 24333650

[ref17] Candelori C. Ciocca A. (1998). “Attachment and eating disorders.” in Psychotherapeutic issues in eating disorders: Models, methods and results. eds. Bria A. Ciocca A. Risio S. De (Roma: Società Editrice Universo), 139–153.

[ref18] Carcione A. Semerari A. Nicolò G. (2016). Curare i casi complessi: la terapia metacognitiva interpersonale dei disturbi di personalità. Gius. Laterza & Figli Spa.

[ref20] Castiglioni M. Pepe A. Gandino G. Veronese G. (2013). Self-other positioning in obesity: A pilot study using repertory grid technique. Open Psychol. J. 6, 61–68. doi: 10.2174/1874350101306010061

[ref21] Cole-Detke H. Kobak R. (1996). Attachment processes in eating disorder and depression. J. Consult. Clin. Psychol. 64, 282–290. doi: 10.1037//0022-006x.64.2.282, PMID: 8871412

[ref22] Compare A. Maxwell H. Brugnera A. Zarbo C. Dalle Grave R. Tasca G. A. (2018). Change in attachment dimensions and reflective functioning following emotionally focused group therapy for binge eating disorder. Int. J. Group Psychother. 68, 385–406. doi: 10.1080/00207284.2018.1429928 38449136

[ref23] Cook-Cottone C. (2006). The attuned representation model for the primary prevention of eating disorders: An overview for school psychologists. Psychol. Sch. 43, 223–230. doi: 10.1002/(ISSN)1520-6807

[ref24] Crittenden P. M. (1992). Quality of attachment in the preschool years. Dev. Psychopathol. 4, 209–241. doi: 10.1017/S0954579400000110

[ref25] Davila J. Cobb R. J. (2003). Predicting change in self-reported and interviewer-assessed adult attachment: tests of the individual difference and life stress models of attachment change. Personal. Soc. Psychol. Bull. 29, 859–870. doi: 10.1177/0146167203029007005, PMID: 15018674

[ref26] De Campora G. Larciprete G. Delogu A. M. Meldolesi C. Giromini L. (2016). A longitudinal study on emotional dysregulation and obesity risk: From pregnancy to 3 years of age of the baby. Appetite 96, 95–101. doi: 10.1016/j.appet.2015.09.012, PMID: 26375359

[ref27] DeKlyen M. Greenberg M. T. (2016). “Attachment and psychopathology in childhood.” in Handbook of Attachment: Theory, Research, and Clinical Applications. eds. Cassidy J. Shaver P. R. (New York: The Guilford Press), 637–665.

[ref28] Delvecchio E. Di Riso D. Salcuni S. Lis A. George C. (2014). Anorexia and attachment: dysregulated defense and pathological mourning. Front. Psychol. 5:1218. doi: 10.3389/fpsyg.2014.01218, PMID: 25389412 PMC4211560

[ref30] Di Fini G. Civilotti C. Zaccagnino M. Veglia F. (2013). Attaccamento Adulto e Temi di Vita: una ricerca qualitativa attraverso l’analisi testuale delle Adult Attachment Interview. Quad. di Psicoter. Cogn. 32, 45–60. doi: 10.3280/QPC2013-032004

[ref31] Di Fini G. Veglia F. (2019). Life themes and attachment system in the narrative self-construction: direct and indirect indicators. Front. Psychol. 10:1393. doi: 10.3389/fpsyg.2019.01393, PMID: 31275207 PMC6591274

[ref130] Enckell H. (2002). Metaphor and Psychodynamic Functions of the Mind. Finland: Kuopion yliopisto.

[ref32] Fairburn C. G. Harrison P. J. (2003). Eating disorders. Lancet 361, 407–416. doi: 10.1016/S0140-6736(03)12378-1 12573387

[ref33] Farstad S. M. McGeown L. M. von Ranson K. M. (2016). Eating disorders and personality, 2004–2016: A systematic review and meta-analysis. Clin. Psychol. Rev. 46, 91–105. doi: 10.1016/j.cpr.2016.04.005

[ref34] Favaro A. Zanetti T. Tenconi E. Degortes D. Ronzan A. Veronese A.. (2004). The relationship between temperament and impulsive behaviors in eating disordered subjects. Eat. Disord. 13, 61–70. doi: 10.1080/10640260590893647, PMID: 16864331

[ref35] Felitti V. J. Anda R. F. Nordenberg D. Williamson D. F. Spitz A. M. Edwards V.. (1998). Relationship of childhood abuse and household dysfunction to many of the leading causes of death in adults: the adverse childhood experiences (ACE) study. Am. J. Prev. Med. 14, 245–258. doi: 10.1016/S0749-3797(98)00017-8, PMID: 9635069

[ref36] Foa E. B. Feske U. Burdock T. B. Kozak M. J. McCarthy P. R. (1991). Processing of threat-related information in rape victims. J. Abnorm. Psychol. 100, 156–162. doi: 10.1037//0021-843x.100.2.156, PMID: 2040766

[ref37] Fogel T. F. A. (1982). Emotion and Early Interaction. United Kingdom: Psychology Press.

[ref132] Fonagy P. Target M. (2001). Attaccamento e Funzione Riflessiva Selected papers of Peter Fonagy and Mary Target. Milano: Raffaello Cortina.

[ref38] Fonagy P. Gergely G. Jurist E. L. Target M. (Eds.). (2002). Affect Regulation, Mentalization and the Development of the Self. New York: Other Press.

[ref39] Fonagy P. Leigh T. Steele M. Steele H. Kennedy R. Mattoon G.. (1996). The relation of attachment status, psychiatric classification, and response to psycho- therapy. J. Consult. Clin. Psych. 64, 22–31. doi: 10.1037//0022-006x.64.1.22, PMID: 8907081

[ref41] Fuchs T. (2021). The disappearing body: anorexia as a conflict of embodiment. Eating Weight Disorders-Stud. Anorexia, Bulimia Obesity, 1-9, doi:10.1007/s40519-021-01122-7 PMID: 33666885 PMC8860785

[ref42] Gazzaniga M. S. (2006). Handbook of Functional Neuroimaging of Cognition. Cambridge, MA: MIT Press.

[ref43] Gazzaniga M. S. (2011). Neuroscience in the courtroom. Sci. Am. 304, 54–59. doi: 10.1038/scientificamerican0411-34 21495482

[ref44] George C. Kaplan N. Main M. (1996). Adult attachment interview. Unpublished Manuscript. University of California, Berkeley, 72,

[ref45] Gordon I. Voos A. C. Bennett R. H. Bolling D. Z. Pelphrey K. A. Kaiser M. D. (2013). Brain mechanisms for processing affective touch. Hum. Brain Mapp. 34, 914–922. doi: 10.1002/hbm.21480, PMID: 22125232 PMC6869848

[ref46] Graves T. A. Tabri N. Thompson-Brenner H. Franko D. L. Eddy K. T. Bourion-Bedes S., . & Thomas J. J. (2017). A meta-analysis of the relation between therapeutic alliance and treatment outcome in eating disorders. Int. J. Eat. Disord., 50, 323–340. doi: 10.1002/eat.22672, PMID: 28152196

[ref47] Grenon R. Tasca G. A. Maxwell H. Balfour L. Proulx G. Bissada H. (2016). Parental bonds and body dissatisfaction in a clinical sample: the mediating roles of attachment anxiety and media internalization. Body Image 19, 49–56. doi: 10.1016/j.bodyim.2016.08.005, PMID: 27614193

[ref48] Guidano V. F. (1988). La complessità del Sé: un approccio sistemico-processuale alla psicopatologia e alla terapia cognitiva. Turin: Bollati Boringhieri.

[ref49] Harrison A. O'Brien N. Lopez C. Treasure J. (2010). Sensitivity to reward and punishment in eating disorders. Psychiatry Res. 177, 1–11. doi: 10.1016/j.psychres.2009.06.010 20381877

[ref50] Hofer M. A. (2006). Psychobiological roots of early attachment. Curr. Dir. Psychol. Sci. 15, 84–88. doi: 10.1111/j.0963-7214.2006.00412.x

[ref51] Hughes S. O. Power T. G. Papaioannou M. A. Cross M. B. Nicklas T. A. Hall S. K.. (2011). Emotional climate, feeding practices, and feeding styles: an observational analysis of the dinner meal in head start families. Int. J. Behav. Nutr. Phys. Activity 8:1e11. doi: 10.1186/1479-5868-8-60 PMC3129575 21663653

[ref52] Illing V. Tasca G. A. Balfour L. Bissada H. (2010). Attachment insecurity predicts eating disorder symptoms and treatment outcomes in a clinical sample of women. J. Nerv. Ment. Dis. 198, 653–659. doi: 10.1097/NMD.0b013e3181ef34b2 20823727

[ref54] Johnson C. Connors M. E. (1987). The Etiology and Treatment of Bulimia Nervosa: A Biopsychosocial Perspective. New York: Basic Books.

[ref55] Keating L. Tasca G. A. Hill R. (2013). Structural relationships among attachment inse- curity, alexithymia, and body esteem in women with eating disorders. Eat. Behav. 14, 366–373. doi: 10.1016/j.eatbeh.2013.06.013, PMID: 23910782

[ref56] Krystal H. (1978). Self representation and the capacity for self care. Annu. Psychoanal. 10, 43–65. doi: 10.1007/BF02109778

[ref57] Kuipers G. S. Bekker M. H. J. (2012). Attachment, mentalization and eating disorders: a review of studies using the adult attachment interview. Curr. Psychiatr. Rev. 8, 326–336. doi: 10.2174/157340012803520478

[ref58] Lanius R. A. Bluhm R. L. Frewen P. A. (2011). How understanding the neurobiology of complex post-traumatic stress disorder can inform clinical practice: A social cognitive and affective neuroscience approach. Acta Psychiatr. Scand. 124, 331–348. doi: 10.1111/j.1600-0447.2011.01755.x, PMID: 21854369

[ref59] Latzer Y. Hochdorf Z. Bachar E. Canetti L. (2002). Attachment style and family functioning as discriminating factors in eating disorders. Contemp. Fam. Ther. 24, 581–599. doi: 10.1023/A:1021273129664

[ref60] Legerstee M. (2005). Infants’ Sense of People: Precursors to a Theory of Mind. Cambridge Cambridge University Press.

[ref61] Legrand D. Briend F. (2015). Anorexia and bodily intersubjectivity. Eur. Psychol. 20, 52–61. doi: 10.1027/1016-9040/a000208

[ref134] Liotti G. (2001). Le opere della coscienza. Milano: Raffaello Cortina.

[ref62] Liotti G. (2004). Trauma, dissociation, and disorganized attachment: three strands of a single braid. Psychother. Theory Res. Pract. Train. 41, 472–486. doi: 10.1037/0033-3204.41.4.472

[ref63] Liotti G. Monticelli F. (2008). I sistemi motivazionali nel dialogo clinico. Milano: Raffaello Cortina Editore.

[ref64] Liotti G. Monticelli F. (2014). Teoria e clinica dell’alleanza terapeutica. Una prospettiva cognitivo-evoluzionista. Milano: Raffaello Cortina Editore.

[ref65] Lunn S. Poulsen S. Daniel S. I. F. (2012). Subtypes in bulimia nervosa: The role of eat- ing disorder symptomatology, negative affect, and interpersonal function- ing. Compr. Psychiatry 53, 1078–1087. doi: 10.1016/j.comppsych.2012.04.005, PMID: 22591731

[ref66] Lyons-Ruth K. Jacobvitz D. (1999). “Attachment disorganization: unresolved loss, relational violence, and lapses in behavioral and attentional strategies.” in Handbook of Attachment: Theory, Research, and Clinical Applications. eds. Cassidy J. Shaver P. R. (New York: The Guilford Press), 520–554.

[ref67] Martinez M. A. Craighead L. W. (2015). Toward person (ality)-centered treatment: how consideration of personality and individual differences in anorexia nervosa may improve treatment outcome. Clin. Psychol. Sci. Pract. 22, 296–314. doi: 10.1037/h0101728

[ref68] Maxwell H. Compare A. Brugnera A. Zarbo C. Rabboni M. Dalle Grave R.. (2018). Reflective functioning and growth in therapeutic alliance during emotionally focused group therapy for binge-eating disorder. Group Dyn. Theory Res. Pract. 22, 32–44. doi: 10.1037/gdn0000078

[ref69] Meteyard L. Cuadrado S. R. Bahrami B. Vigliocco G. (2012). Coming of age: A review of embodiment and the neuroscience of semantics. Cortex 48, 788–804. doi: 10.1016/j.cortex.2010.11.002, PMID: 21163473

[ref70] Mikulincer M. Shaver P. R. (2016). Attachment in Adulthood: Structure, Dynamics, and Change. 2nd ed. New York, NY: Guilford Press.

[ref71] Monteleone A. M. Ruzzi V. Patriciello G. Pellegrino F. Cascino G. Castellini G.. (2020). Parental bonding, childhood maltreatment and eating disorder psychopathology: an investigation of their interactions. Eat. Weight Disord. 25, 577–589. doi: 10.1007/s40519-019-00649-0, PMID: 30734225

[ref72] Muzi L. Tieghi L. Rugo M. A. Lingiardi V. (2021). Personality as a predictor of symptomatic change in a residential treatment setting for anorexia nervosa and bulimia nervosa. Eat. Weight Disord. 26, 1195–1209. doi: 10.1007/s40519-020-01023-1, PMID: 33048329 PMC8062347

[ref73] Nelson K. Fivush R. (2004). The emergence of autobiographical memory: a social cultural developmental theory. Psychol. Rev. 111, 486–511. doi: 10.1037/0033-295X.111.2.486, PMID: 15065919

[ref74] Palmisano G. L. Innamorati M. Vanderlinden J. (2016). Life adverse experiences in relation with obesity and binge eating disorder: A systematic review. J. Behav. Addict. 5, 11–31. doi: 10.1556/2006.5.2016.018, PMID: 28092189 PMC5322988

[ref75] Panksepp J. (2004). Affective Neuroscience: The Foundations of Human and Animal Emotions. Oxford: Oxford University Press.

[ref76] Pellegrini R. Veglia F. (1999). “Procedure e strumenti per la raccolta della storia personale.” in Storie di vita. Narrazione e cura in Psicoterapia cognitiva. ed. Veglia F. (Torino: Bollati Boringhieri), 247–250.

[ref78] Phelps E. A. LeDoux J. E. (2005). Contributions of the amygdala to emotion processing: from animal models to human behavior. Neuron 48, 175–187. doi: 10.1016/j.neuron.2005.09.025, PMID: 16242399

[ref79] Piaget J. (1952). The Origins of Intelligence in Children. New York: International Universities Press.

[ref80] Pignatelli A. M. Wampers M. Loriedo C. Biondi M. Vanderlinden J. (2017). Childhood neglect in eating disorders: A systematic review and meta-analysis. J. Trauma Dissociation 18, 100–115. doi: 10.1080/15299732.2016.1198951, PMID: 27282982

[ref81] Porges S. W. (2011). The Polyvagal Theory: Neurophysiological Foundations of Emotions, Attachment, Communication, and Self-Regulation (Norton Series on Interpersonal Neurobiology). New York: WW Norton & Company.

[ref82] Powell E. M. Frankel L. A. Umemura T. Hazen N. (2017). The relationship between adult attachment orientation and child self-regulation in eating: The mediating role of persuasive-controlling feeding practices. Eat. Behav. 26, 121–128. doi: 10.1016/j.eatbeh.2017.02.006, PMID: 28236740

[ref83] Ramacciotti A. Sorbello M. Pazzagli A. Vismara L. Mancone A. Pallanti S. (2001). Attachment processes in eating disorders. Eat. Weight Disord. 6, 166–170. doi: 10.1007/bf03339766 11589419

[ref84] Ringer F. Crittenden P. (2007). Eating disorders and attachment: The effects of hidden family processes on eating disorders. Eur. Eat. Disord. Rev. 15, 119–130. doi: 10.1002/erv.761, PMID: 17676680

[ref85] Riva G. (2014). Out of my real body: cognitive neuroscience meets eating disorders. Front. Hum. Neurosci. 8:236. doi: 10.3389/fnhum.2014.00236, PMID: 24834042 PMC4018545

[ref86] Rø Ø. Martinsen E. W. Hoffart A. Sexton H. Rosenvinge J. H. (2005). Adults with chronic eating disorders. Two-year follow-up after inpa- tient treatment. Eur. Eating Disord. Rev. 13, 255–263. doi: 10.1002/erv.651

[ref87] Ronga I. Galigani M. Bruno V. Noel J.-P. Gazzin A. Perathoner C.. (2021). Spatial tuning of electrophysiological responses to multisensory stimuli reveals a primitive coding of the body boundaries in newborns. Proc. Natl. Acad. Sci. 118:e2024548118. doi: 10.1073/pnas.2024548118, PMID: 33798099 PMC8000027

[ref88] Rothschild-Yakar L. Levy-Shiff R. Fridman-Balaban R. Gur E. Stein D. (2010). Mentali- zation and relationships with parents as predictors of eating disordered behavior. J. Nerv. Ment. Dis. 198, 501–507. doi: 10.1097/NMD.0b013e3181e526c8, PMID: 20611053

[ref91] Schore A. N. (1994). Affect Regulation and the Origin of the Self. Lawrence Erlbaum Associates. Hillsdale, NJ.

[ref135] Schore A. N. (2001). Effects of a secure attachment relationship on right brain development, affect regulation, and infant mental health. Infant Mental Health J. 22, 7–66. doi: 10.1002/1097-0355(200101/04)22:1<7::AID-IMHJ2>3.0.CO;2-N

[ref92] Schore A. N. (2003). Affect Regulation and the Repair of the Self (Norton Series on Interpersonal Neurobiology). New York: WW Norton & Company.

[ref136] Schore J. R. Schore A. N. (2008). Modern attachment theory: The central role of affect regulation in development and treatment. Clin. Soc. Work J. 36, 9–20. doi: 10.1007/s10615-007-0111-7

[ref93] Selvini Palazzoli M. (1988). L’anoressia mentale in una prospettiva sistemica. Psicobiettivo 8, 37–51.

[ref94] Setiadi V. T. Risnawaty W. (2021). “Correlation between thin-ideal pressure and body appreciation Among young adult women,” in *International Conference on Economics, Business, Social, and Humanities (ICEBSH 2021)*. Malaysia: Atlantis Press, 1013–1018.

[ref95] Siegel D. J. (1999). The Developing Mind: Toward a Neurobiology of Interpersonal Experience. New York: Guilford Press.

[ref96] Siegel D. J. (2001). Toward an interpersonal neurobiology of the developing mind: Attachment relationships,“mindsight,” and neural integration. Infant Ment. Heal. J. 22, 67–94. doi: 10.1002/1097-0355(200101/04)22:13.0.CO;2-G

[ref97] Siegel D. (2014). Brainstorm: The Power and Purpose of the Teenage Brain-an Insite-out Guide to the Emerging Adolescent Mind Ages 12–24. Brunswick. Victoria Scr. Publ. Pty Ltd.

[ref98] Skårderud F. (2007). Eating one’s words, part II: The embodied mind and reflective function in anorexia nervosa--theory. Eur. Eat. Disord. Rev. 15, 243–252. doi: 10.1002/erv.778 17676695

[ref99] Slade A. Grienenberger J. Bernbach E. Levy D. Locker A. (2005). Maternal reflective functioning, attachment, and the transmission gap: A preliminary study. Attachment Hum. Dev. 7, 283–298. doi: 10.1080/14616730500245880, PMID: 16210240

[ref101] Spitz R. A. (1958). On the genesis of superego components. Psychoanal. Study Child 13, 375–404. doi: 10.1080/00797308.1958.11823188, PMID: 13614594

[ref102] Stern D. (1985). The Interpersonal World of the Infant. New York: Basic Books.

[ref103] Stice E. Fairburn C. G. (2003). Dietary and dietary–depressive subtypes of bulimia nervosa show differential symptom presentation, social impairment, comor- bidity, and course of illness. J. Consult. Clin. Psych. 71, 1090–1094. doi: 10.1037/0022-006X.71.6.1090, PMID: 14622085

[ref104] Tasca G. A. (2019). Attachment and eating disorders: a research update. Curr. Opin. Psychol. 25, 59–64. doi: 10.1016/j.copsyc.2018.03.003, PMID: 29579723

[ref137] Tasca G. A. Balfour L. (2014). Attachment and eating disorders: A review of current research. Int. J. Eating Disord. 47, 710–717. 10.1002/eat.22302 24862477

[ref105] Tasca G. A. Ritchie K. Zachariades F. Proulx G. Trinneer A. Balfour L.. (2013). Attachment insecurity mediates the relationship between childhood trauma and eating disorder psychopathology in a clinical sample: a structural equation model. Child Abuse Negl. 37, 926–933. doi: 10.1016/j.chiabu.2013.03.004, PMID: 23623443

[ref106] Taylor C. B. Bryson S. Doyle A. A. C. Luce K. H. Cunning D. Abascal L. B.. (2006a). The adverse effect of negative comments about weight and shape from family and siblings on women at high risk for eating disorders. Pediatrics 118, 731–738. doi: 10.1542/peds.2005-1806, PMID: 16882830

[ref107] Taylor G. J. Parker J. D. Bagby R. M. Bourke M. P. (2006b). Relationships between alexithymia and psychological characteristics associated with eating disorders. J. Psychosomatic Res. XLI 41, 561–568. doi: 10.1016/S0022-3999(96)00224-3 9032719

[ref108] Tenconi E. Santonastaso P. Degortes D. Bosello R. Titton F. Mapelli D.. (2010). Set-shifting abilities, central coherence, and handedness in anorexia nervosa patients, their unaffected siblings and healthy controls: exploring putative endophenotypes. World J. Biol. Psychiatry 11, 813–823. doi: 10.3109/15622975.2010.483250, PMID: 20486870

[ref109] Troisi A. Di Lorenzo G. Alcini S. Nanni R. C. Di Pasquale C. Siracusano A. (2006). Body dissatisfaction in women with eating disorders: relationship to early separation anxiety and insecure attachment. Psychosom. Med. 68, 449–453. doi: 10.1097/01.psy.0000204923.09390.5b, PMID: 16738078

[ref110] Tronick E. Z. (1989). Emotions and emotional communication in infants. Am. Psychol. 44, 112–119. doi: 10.1037/0003-066X.44.2.112 2653124

[ref111] Tronick E. Beeghly M. (2011). Infants’ meaning-making and the development of mental health problems. Am. Psychol. 66, 107–119. doi: 10.1037/a0021631, PMID: 21142336 PMC3135310

[ref112] Tronick E. Z. Bruschweiler-Stern N. Harrison A. M. Lyons-Ruth K. Morgan A. C. Nahum J. P., . & Stern D. N. (1998). Dyadically expanded states of consciousness and the process of therapeutic change. Infant Mental Health J., 19, 290–299. doi: 10.1002/(SICI)1097-0355(199823)19:3<290::AID-IMHJ4>3.0.CO;2-Q

[ref113] Ugazio V. (2013). Semantic Polarities and Psychopathologies in the Family: Permitted and Forbidden Stories. London: Routledge.

[ref114] Van der Kolk B. (2014). The Body Keeps the Score: Mind, Brain and Body in the Transformation of Trauma. London: Penguin UK.

[ref115] Veglia F. (1999). Storie di vita. Narrazione e cura in psicoterapia cognitiva. Bollati Boringhieri.

[ref116] Veglia F. (2013). “Narrazione: origine, funzioni e necessità” in Veglia, Narrazione e disabilità intellettiva. eds. Ruggerini S. C. Manzotti G. Griffo F. (Trento: Centro Studi Erickson), 43–62.

[ref117] Veglia F. Di Fini G. (2017). Life themes and interpersonal motivational systems in the narrative self-construction. Front. Psychol. 8:1897. doi: 10.3389/fpsyg.2017.01897, PMID: 29163279 PMC5664818

[ref118] Veglia F. Finzi S. Civilotti C. Di Fini G. (2019). Evoluzione della CBT. Fondamenti e Implicazioni Neurobiologiche. cited in Pagani, M., and Carletto, S. (2019). Il cervello che cambia: Neuroimaging: il contributo alle neuroscienze. Sesto San Giovanni: Mimesis, 255–290.

[ref119] Verschueren M. Claes L. Palmeroni N. Raemen L. Buelens T. Moons P.. (2021). Identity functioning and eating disorder symptomatology: The role of cognitive emotion regulation strategies. Front. Psychol. 12:667235. doi: 10.3389/fpsyg.2021.667235, PMID: 34122260 PMC8194491

[ref120] Vrabel K. R. Rosenvinge J. H. Hoffart A. Martinsen E. W. Ro O. (2008). The course of illness following inpatient treatment of adults with longstanding eating disorders: A 5-year follow-up. Int. J. Eat. Disord. 119, 623–629. doi: 10.1037/a0019857 18176949

[ref121] Ward A. Ramsay R. Turnbull S. Steele M. Steele H. Treasure J. (2001). Attachment in anorexia nervosa: a transgenerational perspective. Br. J. Med. Psychol. 74, 497–505. doi: 10.1348/000711201161145, PMID: 11780797

[ref122] Wei M. Russell D. W. Zakalik R. A. (2005). Adult attachment, social self-efficacy, self-disclosure, loneliness, and subsequent depression for freshman college students: A longitudinal study. J. Couns. Psychol. 52:602, –614. doi: 10.1037/0022-0167.52.4.602

[ref124] Wierenga C. E. Ely A. Bischoff-Grethe A. Bailer U. F. Simmons A. N. Kaye W. H. (2014). Are extremes of consumption in eating disorders related to an altered balance between reward and inhibition? Front. Behav. Neurosci. 8:410. doi: 10.3389/fnbeh.2014.00410, PMID: 25538579 PMC4260511

[ref125] Wright K. (1991). Vision and separation: Between mother and baby. NJ: Jason Aronson.

[ref126] Zachrisson H. D. Kulbotten G. R. (2006). Attachment in anorexia nervosa: An exploration of associations with eating disorder psychopathology and psychiatric symptoms. Eat. Weight Disord. 11, 163–170. doi: 10.1007/BF03327567, PMID: 17272945

[ref127] Zachrisson H. D. Skårderud F. (2010). Feelings of insecurity: review of attachment and eating disorders. Eur. Eat. Disord. 18, 97–106. doi: 10.1002/erv.999, PMID: 20148392

[ref128] Zaitsoff S. Pullmer R. Cyr M. Aime H. (2015). The role of the therapeutic alliance in eating disorder treatment outcomes: a systematic review. Eat. Disord. 23, 99–114. doi: 10.1080/10640266.2014.964623, PMID: 25330409

